# Supplementary material for: Different gut microbial communities correlate with efficacy of albendazole-ivermectin against soil-transmitted helminthiases
Source: Nat Commun. 2022 Feb 25;13:1063. doi: 10.1038/s41467-022-28658-1 (PMC8881608; doi:10.1038/s41467-022-28658-1)
Supplement: Supplementary file 1 — Supplementary information file [file 41467_2022_28658_MOESM1_ESM.pdf]

**Supplementary information to the manuscript:**

**Different gut microbial communities correlate with efficacy of albendazole-ivermectin against soil-transmitted helminthiases**

Pierre H.H. Schneeberger[1,2], Morgan Gueuning[3], Sophie Welsche[1,2], Eveline Hürlimann[1,2], Julian Dommann[1,2], Cécile Häberli[1,2], Jürg E. Frey[3], Somphou Sayasone[2,4], Jennifer Keiser[1,2]

1 University of Basel, Basel, Switzerland, 2 Swiss Tropical and Public Health Institute, Department of Medical Parasitology and Infection Biology, Helminth Drug Development Unit, Basel, Switzerland, 3 Agroscope, Research Group Molecular Diagnostics, Genomics and Bioinformatics, Wädenswil, Switzerland, 4 Lao Tropical and Public Health Institute, Vientiane, Lao People's Democratic Republic

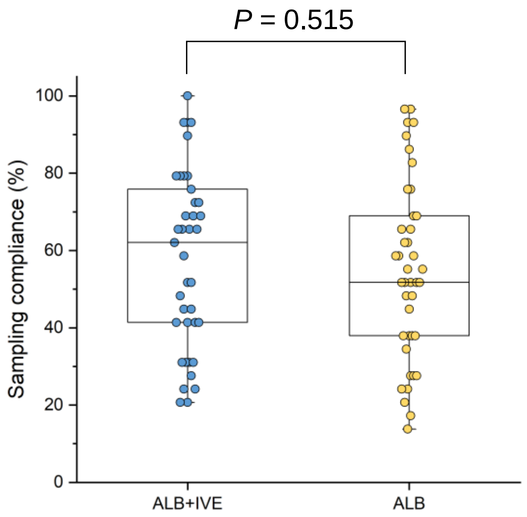


**Supplementary figure 1.** Sampling compliance, by treatment arm. A two-sided Mann-Whitney test was used to compare sampling compliance between the monotherapy arm (n=41) and the combination therapy arm (n=39). The lower and upper bound of each box represent the 25th and 75th percentiles, respectively, and the line within indicates the median. The whiskers represent the minimum and maximum values.

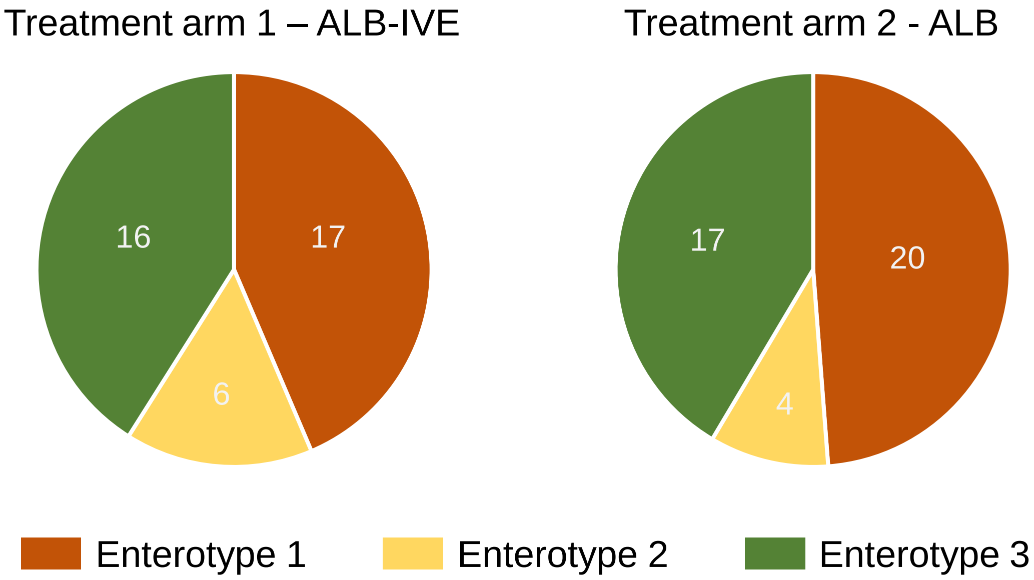


**Supplementary figure 2.** Proportion of each enterotype, by treatment arm. ALB = albendazole; IVE = ivermectin.

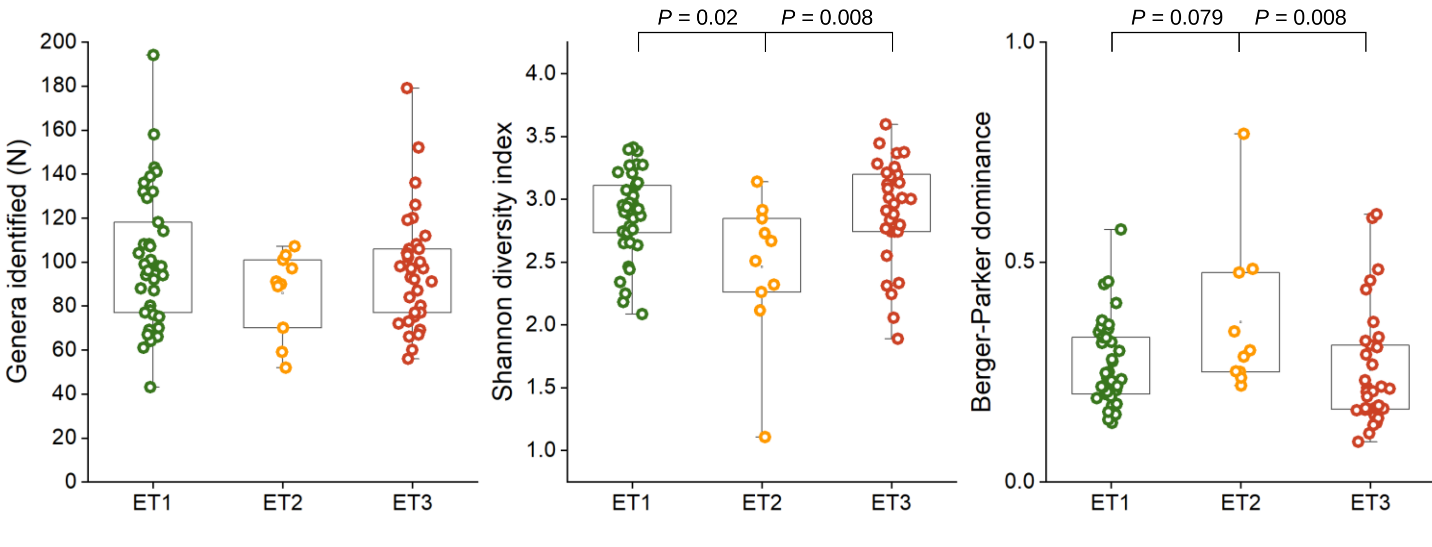


**Supplementary figure 3.** Alpha diversity indices, by enterotype. Group comparison between enterotype 1 (ET1; n=37), enterotype 2 (ET2; n=10), and enterotype 3 (ET3; n=33) was performed with a Kruskal-Wallis test and adjusted for multiple testing bias using the Bonferroni procedure. The lower and upper bound of each box represent the 25th and 75th percentiles, respectively, and the line within indicates the median. The whiskers represent the minimum and maximum values.

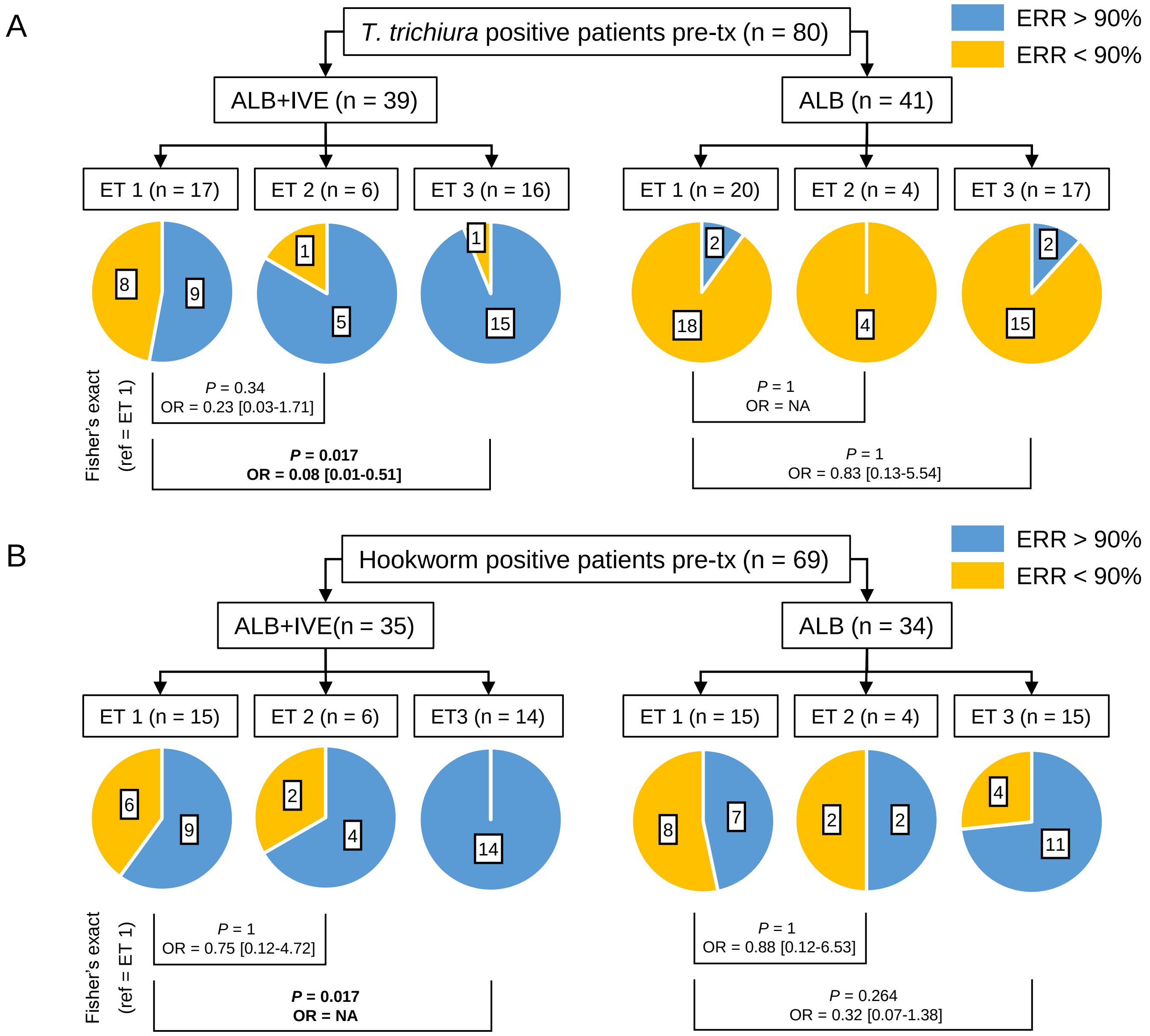


**Supplementary figure 4.** Association between egg reduction rate (ERR) and pre-treatment enterotype (ET), by treatment arm. A. Association between treatment outcome of *Trichuris trichiura* and ET at baseline. **B.** Association measured between treatment outcome of hookworm and ET before treatment. Egg reduction rate is the ratio of average eggs per gram (EPG) of stool between days 14-28 post-treatment and pre-treatment EPG. The threshold for treatment success is an ERR equal or above 90%. The Fisher's exact tests were two-sided and the 95% confidence interval of the odds ratios is shown in bracket. Labels on the pie charts represent the number of patients in each group. n = number of patients; OR = odds ratios.

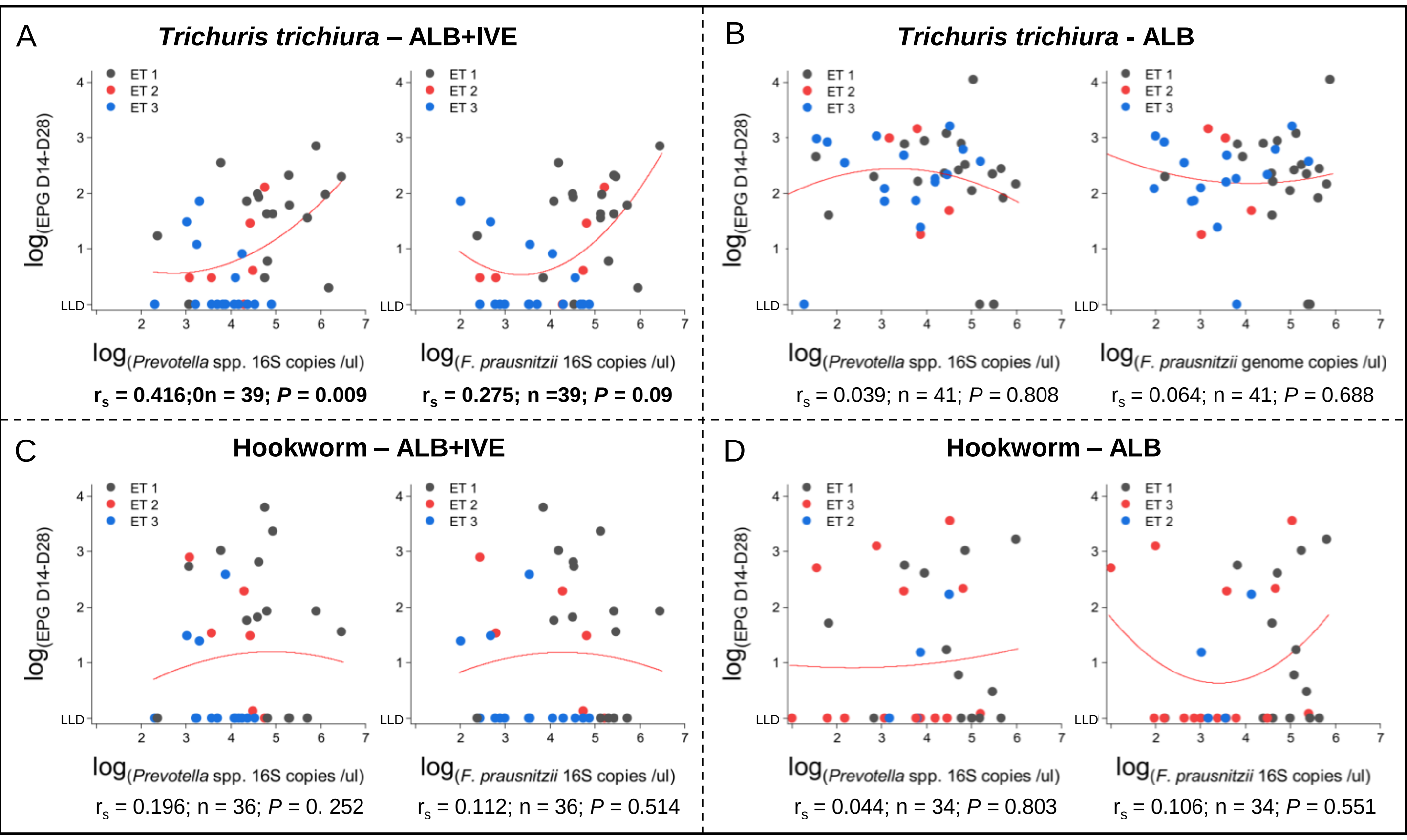


**Supplementary figure 5.** Correlation between average eggs per gram of stool counts from days 14 to 28 and copy number of *Prevotella* and *Faecalibacterium prausnitzii,* measured by qPCR. Significance of the Spearman correlation was calculated using a two-sided test. $r_s$ = Spearman correlation coefficient; EPG = eggs per gram of stool; ET(1-3) = enterotypes 1 to 3; ALB+IVE = albendazole and ivermectin; ALB = albendazole.

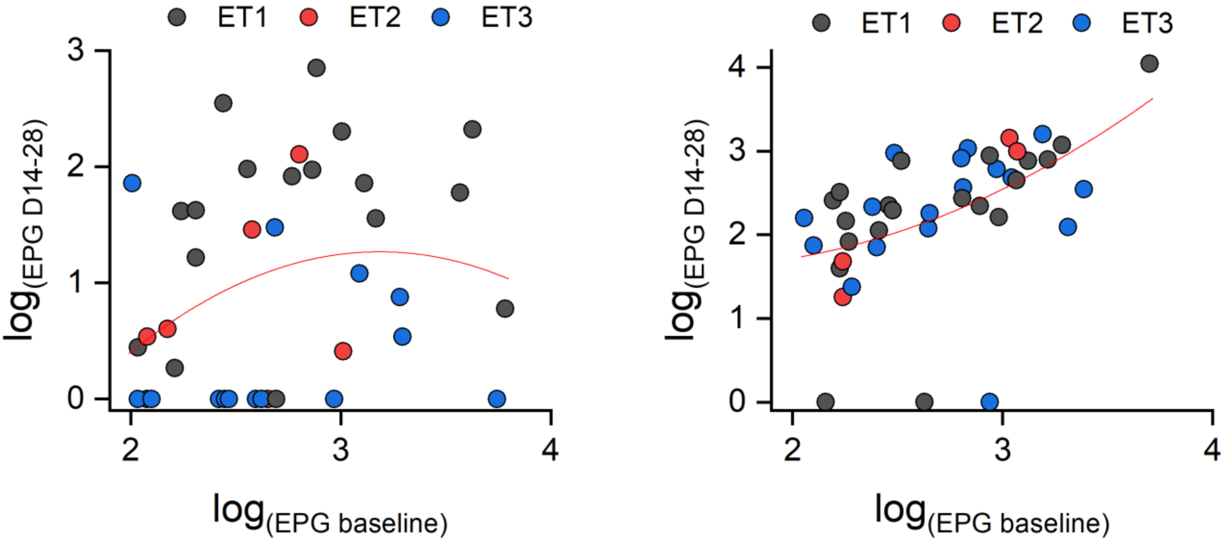


$r_s = 0.257$; n = 39; $P = 0.115$

**$r_s = 0.633$; n = 41; $P < 0.0001$**

**Supplementary figure 6.** Spearman correlation between average *T. trichiura* eggs per gram (EPG) of stool counts from days 14 to 28 and baseline (pre-treatment) EPG, by treatment arm. The left half includes patients who received albendazole and ivermectin while the right half includes patients who received only albendazole. Significance of the Spearman correlation was calculated using a two-sided test. $r_s$ = Spearman correlation coefficient; EPG = eggs per gram of stool.

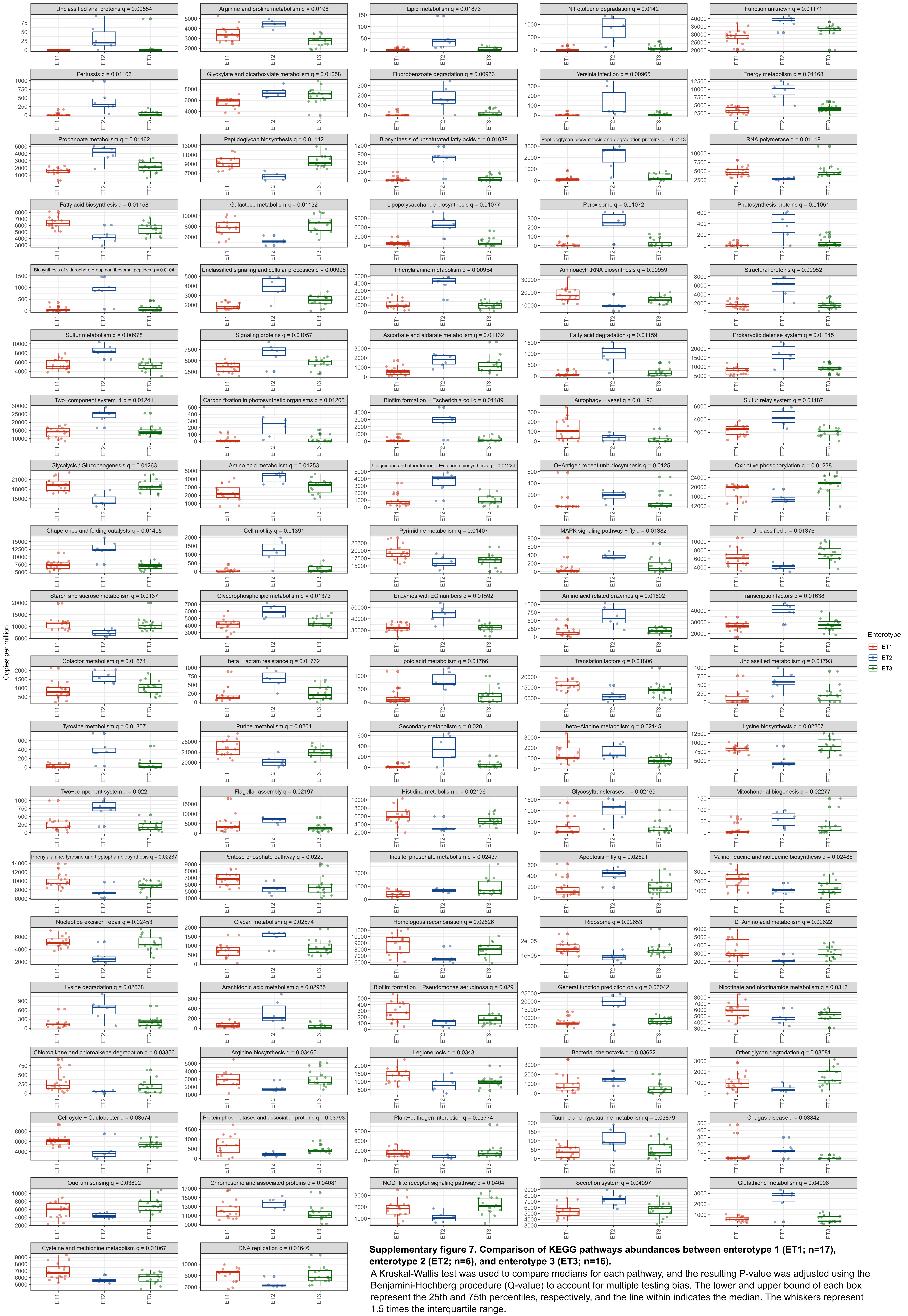


**Supplementary figure 7. Comparison of KEGG pathways abundances between enterotype 1 (ET1; n=17), enterotype 2 (ET2; n=6), and enterotype 3 (ET3; n=16).**
A Kruskal-Wallis test was used to compare medians for each pathway, and the resulting P-value was adjusted using the Benjamini-Hochberg procedure (Q-value) to account for multiple testing bias. The lower and upper bound of each box represent the 25th and 75th percentiles, respectively, and the line within indicates the median. The whiskers represent 1.5 times the interquartile range.

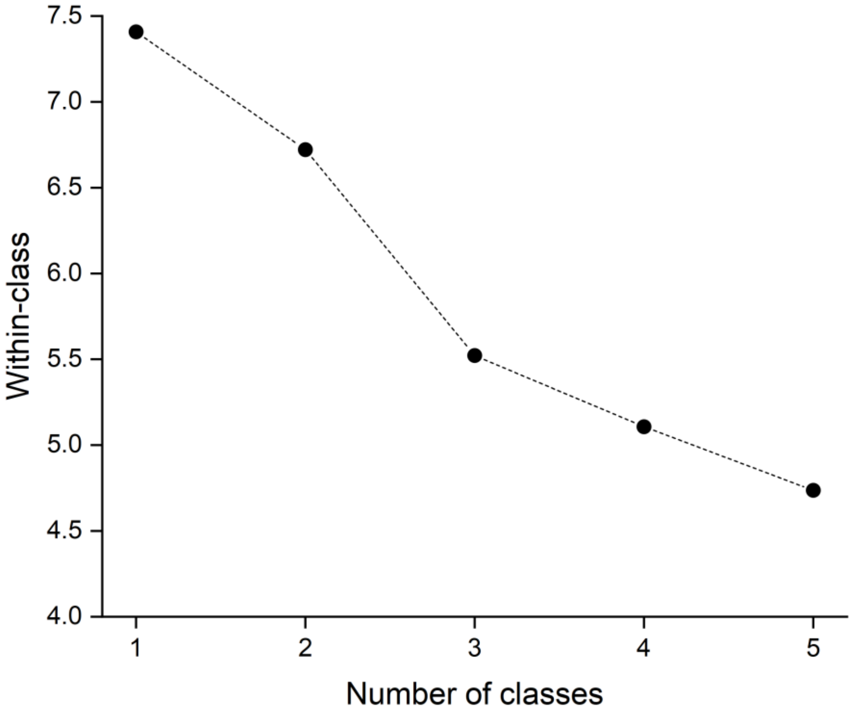


**Supplementary figure 8.** Evolution of inertia during k-means clustering.

**Supplementary table 1**. Shotgun sequencing depth.

| Patient_ID | Shotgun sequencing depth (M sequences) |
|---|---|
| 8764 | 8.5 |
| 8842 | 7 |
| 8946 | 13.3 |
| 8985 | 10 |
| 8999 | 5.5 |
| 9023 | 6 |
| 9951 | 5.6 |
| 9952 | 6.3 |
| 9959 | 11.4 |
| 9960 | 5.6 |
| A0872 | 7.6 |
| A0875 | 7.9 |
| A0885 | 4.6 |
| A0944 | 6.8 |
| A1001 | 9.4 |
| A1025 | 7.2 |
| A1032 | 9.6 |
| A1033 | 7.9 |
| A1042 | 7.4 |
| A1073 | 5.2 |
| A1091 | 8.4 |
| A1172 | 4.4 |
| A1174 | 9.6 |
| A1191 | 6.4 |
| A1221 | 10.3 |
| A1242 | 6.3 |
| A1244 | 7.7 |
| A1261 | 6.7 |
| A1272 | 7.9 |
| A1276 | 8.6 |
| A1291 | 9.9 |
| A1301 | 5.5 |
| A1332 | 5 |
| A1341 | 5.3 |
| A1344 | 6.6 |
| A1362 | 5.7 |
| A1382 | 5.3 |
| A1393 | 6.5 |
| A1482 | 7.3 |

**Supplementary table 2.** Spearman correlation between community metrics and sequencing depth to assess bias related to low sequencing coverage. A value in bold indicates a significant correlation ($P < 0.05$) measured using a two-sided test.

| | | | Taxonomic features | | | Functional features | |
|---|---|---|---|---|---|---|---|
| | **Spearman correlation matrix** | Sequencing depth ($log10_{sequenced\ reads}$) | Taxa (N) | Shannon diversity | Berger-Parker dominance | Number of Kegg Orthology terms identified | Complete metabolic pathways (>99% confidence) |
| | Sequencing depth ($log10_{sequenced\ reads}$) | **1** | 0.097 | -0.243 | 0.091 | 0.057 | 0.149 |
| **Taxonomic features** | Taxa (N) | 0.097 | **1** | **0.551** | **-0.425** | 0.089 | 0.189 |
| | Shannon diversity | -0.243 | **0.551** | **1** | **-0.853** | -0.051 | **0.322** |
| | Berger-Parker dominance | 0.091 | **-0.425** | **-0.853** | **1** | 0.188 | -0.278 |
| **Functional features** | Number of Kegg Orthology terms identified | 0.057 | 0.089 | -0.051 | 0.188 | **1** | **0.449** |
| | Complete metabolic pathways (>99% confidence) | 0.149 | 0.189 | **0.322** | -0.278 | **0.449** | **1** |

**Supplementary table 3.** 16S and taxon-specific qPCR primers and conditions

**Bacterial 16S**

| Primer | Primer sequence | Master mix | | | Cycling conditions | |
|---|---|---|---|---|---|---|
| F | 5-TCCTACGGGAGGCAGCAGT-3 | Taqman Mix | 1X | 5 | | ← 45 cycles → |
| R | 5-GGACTACCAGGGTATCTAATCCTGTT-3 | F primer | 0.3 uM | 1 | 95°C | 95°C |
| probe | (FAM)-CGTATTACCGCGGCTGCTGGCAC-(NFQ-MGB) | R primer | 0.3 uM | 1 | 10 min | 15 sec; 60°C |
| | | Probe | 0.2 uM | 1 | | 1 min |
| | | DNA | | 2 | | |

**FAEC**

| Primer | Primer sequence | Master mix | | | Cycling conditions | |
|---|---|---|---|---|---|---|
| F | 5-TGTAAACTCCTGTTGTTGAGGAAGATAA-3 | Taqman Mix | 1X | 5 | | ← 45 cycles → |
| R | 5-GCGCTCCCTTTACACCCA-3 | F primer | 0.3 uM | 1 | 95°C | 95°C |
| probe | (HEX)-CAAGGAAGTGACGGCTAACTACGTGCCAG-(NFQ-MGB) | R primer | 0.3 uM | 1 | 10 min | 15 sec; 60°C |
| | | Probe | 0.2 uM | 1 | | 1 min |
| | | DNA | | 2 | | |

**PREV**

| Primer | Primer sequence | Master mix | | | Cycling conditions | |
|---|---|---|---|---|---|---|
| F | 5-CCAGCCAAGTAGCGTGCA-3 | Taqman Mix | 1X | 5 | | ← 45 cycles → |
| R | 5-TGGACCTTCCGTATTACCGC-3 | F primer | 0.3 uM | 1 | 95°C | 95°C |
| probe | (FAM)-AATAAGGACCGGCTAATTCCGTGCCAG-(NFQ-MGB) | R primer | 0.3 uM | 1 | 10 min | 15 sec; 60°C |
| | | Probe | 0.2 uM | 1 | | 1 min |
| | | DNA | | 2 | | |

**ESCH**

| Primer | Primer sequence | Master mix | | | Cycling conditions | |
|---|---|---|---|---|---|---|
| F | 5-CATGCCGCGTGTATGAAGAA-3 | Taqman Mix | 1X | 5 | | ← 45 cycles → |
| R | 5-CGGGTAACGTCAATGAGCAAA-3 | F primer | 0.3 uM | 1 | 95°C | 95°C |
| probe | (CY5)-TATTAACTTTACTCCCTTCCTCCCCGCTGAA-(NFQ-MGB) | R primer | 0.3 uM | 1 | 10 min | 15 sec; 60°C |
| | | Probe | 0.2 uM | 1 | | 1 min |
| | | DNA | | 2 | | |

**Supplementary table 4.** Correlation of enterotype-relevant taxa at the genus level between 16S rRNA gene sequencing and shallow shotgun sequencing. A value in bold indicates a significant correlation (P < 0.05) measured using a two-sided test.

| Genus | Spearman correlation | *P*-value |
|---|---|---|
| *Prevotella* | **0.512** | **0.001** |
| *Faecalibacterium* | **0.737** | **<0.0001** |
| *Escherichia* | **0.838** | **<0.0001** |
| *Roseburia* | **0.301** | **0.063** |
| *Eubacterium* | **0.468** | **0.003** |
| *Ruminococcus* | **0.361** | **0.025** |
| *Fusicatenibacter* | 0.2 | 0.222 |
| *Veillonella* | **0.664** | **<0.0001** |

**Supplementary table 5.** Spearman correlation of relative abundances of *Prevotella*, *Faecalibacterium*, and *Escherichia* genera between sequencing (16S rRNA gene and shallow shotgun sequencing) and qPCR-derived values (ratio of target density over total bacteria density). A value in bold indicates a significant correlation (P < 0.05) measured using a two-sided test.

| | | measured by qPCR (ratio of target density over 16S density) | | | | | |
|---|---|---|---|---|---|---|---|
| | | *Prevotella* | P-value | *Faecalibacterium* | P-value | *Escherichia* | P-value |
| measured by 16S rRNA gene sequencing | *Prevotella* | **0.538** | **0** | | | | |
| | *Faecalibacterium* | | | **0.639** | **<0.0001** | | |
| | *Escherichia* | | | | | **0.838** | **<0.0001** |
| measured by shallow shotgun sequencing | *Prevotella* | **0.895** | **<0.0001** | | | | |
| | *Faecalibacterium* | | | **0.737** | **<0.0001** | | |
| | *Escherichia* | | | | | **0.965** | **<0.0001** |
