# Supplementary material for: Different gut microbial communities correlate with efficacy of albendazole-ivermectin against soil-transmitted helminthiases
Source: Nat Commun. 2022 Feb 25;13:1063. doi: 10.1038/s41467-022-28658-1 (PMC8881608; doi:10.1038/s41467-022-28658-1)
Supplement: Supplementary file 3 — Reporting Summary [file 41467_2022_28658_MOESM3_ESM.pdf]

nature portfolio

Corresponding author(s): Schneeberger PHH

Last updated by author(s): Jan 23, 2022

Reporting Summary

Nature Portfolio wishes to improve the reproducibility of the work that we publish. This form provides structure for consistency and transparency in reporting. For further information on Nature Portfolio policies, see our Editorial Policies and the Editorial Policy Checklist.

Statistics

For all statistical analyses, confirm that the following items are present in the figure legend, table legend, main text, or Methods section.

n/a	Confirmed	
☐	☒	The exact sample size (*n*) for each experimental group/condition, given as a discrete number and unit of measurement
☐	☒	A statement on whether measurements were taken from distinct samples or whether the same sample was measured repeatedly
☐	☒	The statistical test(s) used AND whether they are one- or two-sided<br>*Only common tests should be described solely by name; describe more complex techniques in the Methods section.*
☐	☒	A description of all covariates tested
☐	☒	A description of any assumptions or corrections, such as tests of normality and adjustment for multiple comparisons
☐	☒	A full description of the statistical parameters including central tendency (e.g. means) or other basic estimates (e.g. regression coefficient) AND variation (e.g. standard deviation) or associated estimates of uncertainty (e.g. confidence intervals)
☐	☒	For null hypothesis testing, the test statistic (e.g. *F*, *t*, *r*) with confidence intervals, effect sizes, degrees of freedom and *P* value noted<br>*Give P values as exact values whenever suitable.*
☒	☐	For Bayesian analysis, information on the choice of priors and Markov chain Monte Carlo settings
☒	☐	For hierarchical and complex designs, identification of the appropriate level for tests and full reporting of outcomes
☐	☒	Estimates of effect sizes (e.g. Cohen's *d*, Pearson's *r*), indicating how they were calculated

Our web collection on statistics for biologists contains articles on many of the points above.

Software and code

Policy information about availability of computer code

Data collection: Microsoft Access database was used to store data resulting from parasitological readings.

Data analysis:
A. Analysis of quantitative PCR results:
Bio-Rad CFX Maestro 2.0 – v.5.0.21.0616
B. Taxonomic profiling of 16S rRNA sequencing data:
QIIME 2 – v.2020.8 with SILVA database v.138
C. Taxonomic profiling of shotgun sequencing data:
Metaphlan3 – v.3.0.7 with CHOCOPhlAn database v.30
Bowtie2 – v.2.4.5
D. Functional profiling of shotgun sequencing data:
Humann3 - v.3.0.0.alpha.3 with included UniProt/Uniref 2019_01 database
E. To identify compositional differences between identified enterotypes:
LefSe – v.1.0
F. To run the following statistical tests: Mann-Whitney's test; Fisher's exact test of independence; Spearman correlation:
Addinsoft XLSTAT 2021 – v.2020.2.3
G. To generate and optimize graphs:
OriginLab Corporation OriginPro 2021 – v9.8.0.200
H. To generate the heat tree figure and identify compositional differences between enterotypes from the shotgun dataset:
MicrobiomeAnalyst platform (https://www.microbiomeanalyst.ca/, version 06/01/2021) with the included metacoder R package v. 0.3.5.001
I. To conduct gene expression analyses:
Deseq2 - v. 1.34.0
J. Further statistical analyses:

March 2021

R - v.3.6.3 with the following packages:
1) To generate measures of β-diversity (Bray-Curtis dissimilarity) and NMDS ordination coordinates:
vegan - v.2.5-6
2) A custom R-script to run group comparisons of abundances of metabolic pathways using Kruskal-Wallis tests. P-values were adjusted for multiple testing bias using the Bonferroni procedure.
3) To run random forest models:
randomForest - v.4.6-14
4) To run survival analysis and cox proportional hazards bivariate models:
survival – v.3.2-13
5) To generate the AUROC curve for the qPCR values
pROC – v.1.16.2

For manuscripts utilizing custom algorithms or software that are central to the research but not yet described in published literature, software must be made available to editors and reviewers. We strongly encourage code deposition in a community repository (e.g. GitHub). See the Nature Portfolio guidelines for submitting code & software for further information.

Data

Policy information about availability of data

All manuscripts must include a data availability statement. This statement should provide the following information, where applicable:

- Accession codes, unique identifiers, or web links for publicly available datasets
- A description of any restrictions on data availability
- For clinical datasets or third party data, please ensure that the statement adheres to our policy

The sequencing data (16S rRNA gene sequencing and shotgun sequencing) generated in this study have been deposited in the NCBI Short Read Archive under accession code PRJNA767599 (https://www.ncbi.nlm.nih.gov/sra/PRJNA767599).

Field-specific reporting

Please select the one below that is the best fit for your research. If you are not sure, read the appropriate sections before making your selection.

☒ Life sciences ☐ Behavioural & social sciences ☐ Ecological, evolutionary & environmental sciences

For a reference copy of the document with all sections, see nature.com/documents/nr-reporting-summary-flat.pdf

Life sciences study design

All studies must disclose on these points even when the disclosure is negative.

Sample size	For each setting, the trial was powered to detect a significant difference in cure rates between the two treatment groups with 90% power at a two-sided 5% significance level, assuming that the cure rate of albendazole for T. trichiura was 30% and of ivermectin–albendazole was 50%. We calculated that 121 participants per group would be sufficient to test the primary hypothesis that ivermectin–albendazole has a higher efficacy against T. trichiura infection than albendazole alone. Taking a potential loss to follow-up of 15% into account, we anticipated that we needed to enrol 143 participants per treatment group. For the daily sampling sub-study, all eligible participants (n = 88) from the village of Pak Khan, in Laos.
Data exclusions	Data of participants with missing baseline and/or with no daily sampling aliquots for microbiome analysis were excluded as described in Figure 1 (Trial profile).
Replication	Parasitological readings (Kato-Katz smears) were done in quadruplicate to ensure accurate estimation of infection as commonly done.<br>Each qPCR reaction was conducted in duplicate, with a reference standard used to assess inter-plate variability. Inter-replicate variability was assessed and the qPCR reaction repeated if variability was above 1.5 cycles.<br>For 16S rRNA gene sequencing, amplification of the V3-V4 region was conducted in triplicate to avoid PCR amplification bias as recommended.<br>No sample was excluded based on technical variability, hence, replication was successful.
Randomization	Study participants eligible for treatment were randomly assigned to one of the treatment arms using a computer-generated stratified block randomization code. The random allocation sequence with varying random blocks of four or eight and stratified by 2 levels of baseline infection intensity (light: <1000 EPG, and moderate plus heavy: ≥ 1000 EPG T. trichiura infections) was provided by a statistician.
Blinding	The clinical trial was double blinded. Study participants and the trial team/researchers conducting the treatment and assessing the outcomes were blinded using repacked tablets including appearance-matched placebos.

Reporting for specific materials, systems and methods

We require information from authors about some types of materials, experimental systems and methods used in many studies. Here, indicate whether each material, system or method listed is relevant to your study. If you are not sure if a list item applies to your research, read the appropriate section before selecting a response.

Materials & experimental systems

n/a	Involved in the study
☒	☐ Antibodies
☒	☐ Eukaryotic cell lines
☒	☐ Palaeontology and archaeology
☒	☐ Animals and other organisms
☐	☒ Human research participants
☐	☒ Clinical data
☒	☐ Dual use research of concern

Methods

n/a	Involved in the study
☒	☐ ChIP-seq
☒	☐ Flow cytometry
☒	☐ MRI-based neuroimaging

Human research participants

Policy information about studies involving human research participants

Population characteristics	Participants aged 6-60 infected with confirmed T. trichiura infections. Additional demographic information was collected, including gender, weight, and height.
Recruitment	The study was carried out in community members aged 6-60 years in areas with moderate to high T. trichiura endemicity (communities with a prevalence ≥ 25%) identified from earlier studies and/or based on experience of the local collaborating teams. The trial was implemented as a community-based study in order to recruit participants from a broad age range. Entire communities with population size of < 1000 inhabitants were included in the trial for pre-screening and identification of T. trichiura cases to avoid potential selection bias of households.
Ethics oversight	The study was approved by the Lao PDR National Ethics Committee for Health Research, Ministry of Health (reference no. 093/NECHR; date of approval 23 October 2018) and the ethics committees in Switzerland: “Ethikkommission Nordwest- und Zentralschweiz” (BASEC Nr Req-2018-00494; date of approval 05 July 2018)

Note that full information on the approval of the study protocol must also be provided in the manuscript.

Clinical data

Policy information about clinical studies

All manuscripts should comply with the ICMJEguidelines for publication of clinical research and a completedCONSORT checklist must be included with all submissions.

Clinical trial registration	NCT03527732
Study protocol	https://clinicaltrials.gov/ct2/show/NCT03527732
Data collection	Sample collection, data collection, and microscopy. Stool samples were collected within the framework of a multi-country randomized controlled trial assessing the efficacy and safety of the drug combination albendazole-ivermectin against T. trichiura and concomitant helminth infections. In the Nambak District where the microbiome sub-study was conducted, Pak Khan village was selected for the daily sample collection for 28 days post-treatment based on previous good compliance and adequate number of participants living there. In detail, individuals (including parents/caregivers of children) interested in participating in the trial were invited to complete the process of informed consent; thereafter, individuals were assessed for study eligibility during screening procedures. Prior to the start of collection, participants were informed of the aim of the daily sample collection, in addition to the consenting and information sessions conducted for the trial. For screening, two stool samples from different days (within a 5 day interval) were collected and transported to the Nambak hospital. For each stool specimen, duplicate Kato-Katz thick smears (41.7mg each) were prepared and read under a microscope for eggs of T. trichiura, A. lumbricoides, hookworm, and O. viverrini by experienced technicians. Participants underwent a pre-treatment physical examination to collect clinical data and treatment took place approximately 1 week after baseline screening.
Outcomes	T. trichiura infection status assessed by Kato-Katz 14-21 days after treatment was the primary endpoint of the trial and the main outcome for efficacy was expressed as CR (i.e. conversion from being egg positive pre-treatment to egg negative post-treatment) and ERR (secondary end point). Secondary endpoints included further infection status with A. lumbricoides, hookworm and S. stercoralis and related efficacy measures and adverse events.
